# Genetic insights into the complexity of premature ovarian insufficiency

**DOI:** 10.1186/s12958-024-01254-2

**Published:** 2024-08-02

**Authors:** Linhang Nie, Xiaojie Wang, Songyuan Wang, Zhidan Hong, Mei Wang

**Affiliations:** 1 Center for Reproductive Medicine, Zhongnan Hospital of Wuhan University, Wuhan, Hubei P.R. China; 2 WuHan University TaiKang Medical School (School of Basic Medical Sciences), Wuhan, Hubei P.R. China; 3 Second Clinical Hospital of WuHan University, Wuhan, Hubei P.R. China; 4 Clinical Medicine Research Center of Prenatal Diagnosis and Birth Health in Hubei Province, Wuhan, Hubei P.R. China; 5 Wuhan Clinical Research Center for Reproductive Science and Birth Health, Wuhan, Hubei P.R. China

## Abstract

Premature Ovarian Insufficiency (POI) is a highly heterogeneous condition characterized by ovarian dysfunction in women occurring before the age of 40, representing a significant cause of female infertility. It manifests through primary or secondary amenorrhea. While more than half of POI cases are idiopathic, genetic factors play a pivotal role in all instances with known causes, contributing to approximately 20–25% of cases. This article comprehensively reviews the genetic factors associated with POI, delineating the primary candidate genes. The discussion delves into the intricate relationship between these genes and ovarian development, elucidating the functional consequences of diverse mutations to underscore the fundamental impact of genetic effects on POI. The identified genetic factors, encompassing gene mutations and chromosomal abnormalities, are systematically classified based on whether the resulting POI is syndromic or non-syndromic. Furthermore, this paper explores the genetic interplay between mitochondrial genes, such as Required for Meiotic Nuclear Division 1 homolog Gene (*RMND1)*, Mitochondrial Ribosomal Protein S22 Gene (*MRPS22*), Leucine-rich Pentapeptide Repeat Gene (*LRPPRC*), and non-coding RNAs, including both microRNAs and Long non-coding RNAs, with POI. The insights provided serve to consolidate and enhance our understanding of the etiology of POI, contributing to establishing a theoretical foundation for diagnosing and treating POI patients, as well as for exploring the mechanisms underlying the disease.

## Background

In April, 2023, the World Health Organization reported that approximately 17.5% of couples of childbearing age worldwide experience infertility. Female factors account for 30–50% of these cases [1–4]. Premature Ovarian Insufficiency (POI) emerges as a significant cause of female infertility. POI is characterized by the cessation of ovarian function in women before the age of 40, leading to amenorrhea and infertility. Diagnostic criteria include a minimum of 4 months of amenorrhea, elevated Follicle-stimulating hormone (FSH) levels exceeding 25 IU/L, and/or reduced estrogen levels persisting for at least 4 weeks, along with the absence of antral follicles in ovarian ultrasound examination [5, 6]. POI symptoms encompass hot flashes, sweating, tension, loss of libido, weakness, dry skin, mucous membrane dryness, reduced bone mineral density, and metabolic disorders, with infertility being the most profound manifestation [7].

**Fig. 1:**
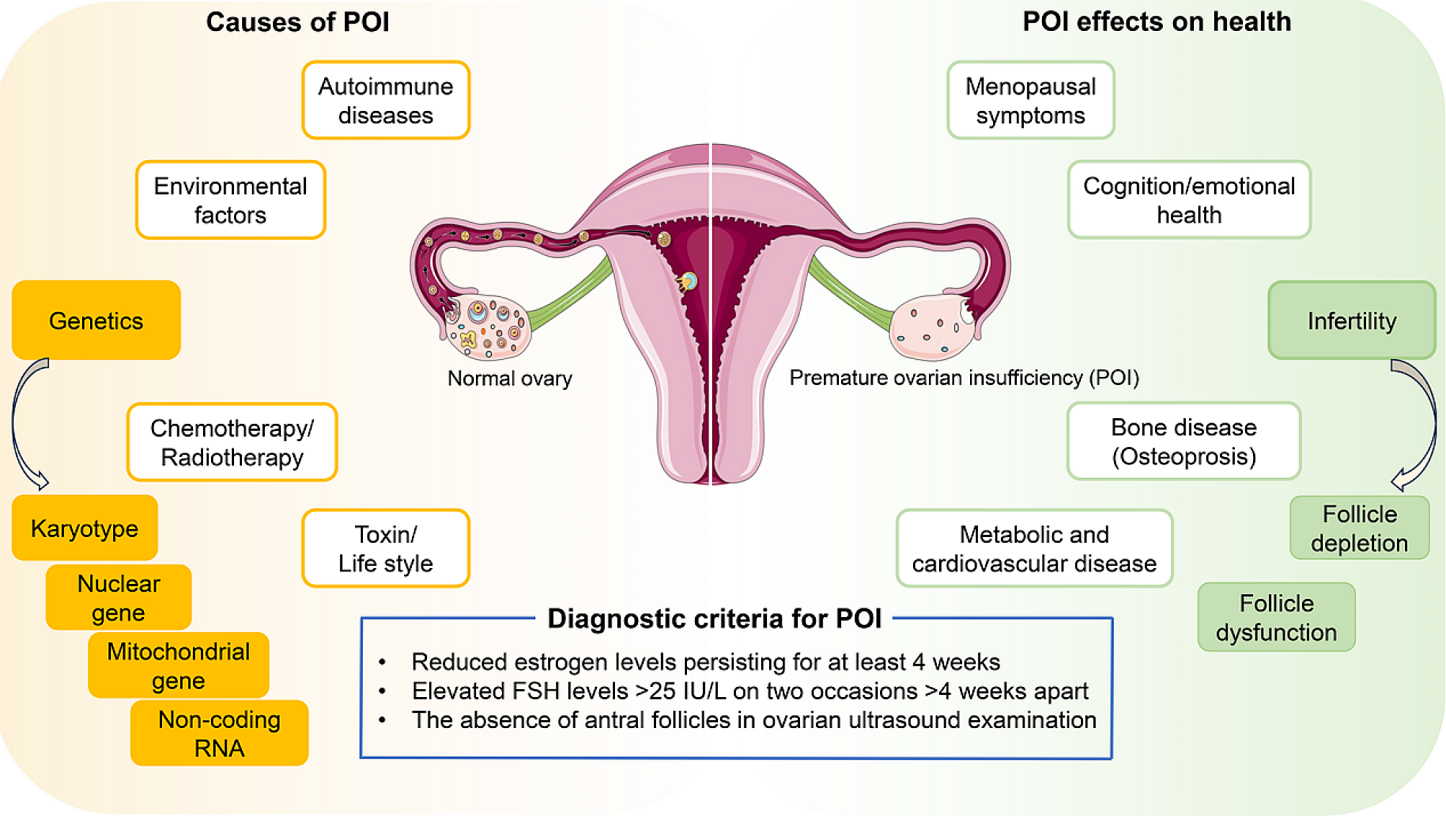
Pathogenic model of premature ovarian insufficiency (POI). Under the regulation of intraovarian factors and gonadotropins, primary follicles develop into preantral and early antral follicles, which are particularly susceptible to atresia, or follicle death. Subsequently, they progress to preovulatory follicles, leading to oocyte release and corpora lutea formation. Defects in folliculogenesis—such as a decrease in primordial follicles, an increase in atresia, and altered follicular maturation—cause POI. Diagnostic criteria for POI include a minimum of four months of amenorrhea, elevated follicle-stimulating hormone (FSH) levels exceeding 25 IU/L, and/or reduced estrogen levels persisting for at least four weeks, alongside the absence of antral follicles on ovarian ultrasound examination. The impact of POI on health is multifaceted, with female infertility primarily resulting from oocyte depletion and dysfunctions in follicular development. The heterogeneity of POI is attributed to genetic causes, including karyotype abnormalities, nuclear genes, mitochondrial genes, and non-coding RNAs, as well as non-genetic causes, such as autoimmunity and environmental toxins

As a heterogeneous condition, POI has various causes, including genetic factors (20–25%), autoimmune factors, iatrogenic influences (both contributing around 10%), and environmental factors (Fig. 1). Nevertheless, the etiology for more than half of patients remains elusive [8].

In a recent study involving 1030 POI patients, 242 cases (23.5%) were identified as associated with the pathogenicity and potential pathogenic mutations of POI-related genes, both known and novel [9]. More than 50 gene mutations associated with POI have been identified, impacting diverse processes, including gonadal development, DNA replication/meiosis, DNA repair, transcription processes, signal transduction, RNA metabolism and translation, and mitochondrial function [9]. Recent studies have unveiled a potential connection between non-coding RNAs (ncRNAs) and POI, such as miRNAs, lnc-RNAs [10–13]. In addition to non-syndrome-associated POI, there are gene variants with POI as a clinical phenotype, and some chromosomal abnormalities contribute to 10–13% of POI cases [14]. Genetic research on these symptomatic POIs is progressively advancing.

This literature review aims to synthesize genetic factors and potential mechanisms related to POI across four dimensions: syndrome-associated POI, non-syndrome POI, mitochondrial dysfunction-associated POI, and non-coding RNA-associated POI. For non-syndrome-associated POI, we categorize the genetic factors based on the roles played by different genes at various stages of follicular development and maturation. Specifically, two novel areas— related to mitochondrial dysfunction-associated POI and non-coding RNA-associated POI— are highlighted to enhance our understanding of POI. This review seek to present current knowledge and hypotheses, identify areas requiring further investigation, and outline future research directions.

## The genetic etiology of syndrome-related POI

### Chromosome abnormality

#### X chromosome aneuploidies

Turner Syndrome (TS) is a genetic disorder arising from the complete or partial absence of an X chromosome, typical ly discerned through a 45, X karyotype. It is characterized by POI, hypogonadism, and short stature [15]. Prevalent in approximately 1 in 2500 live births [16], TS is a significant contributor to POI cases, constituting 4–5% of the total [17]. Female individuals with TS undergo follicle loss during early ovarian development, leading to elevated FSH levels in infancy. This elevation can result in primary amenorrhea and ovarian dysplasia. The underlying mechanism involves the deletion of an X chromosome during meiosis or the loss of crucial X-linked genes, such as the Short-stature homeobox**(***SHOX*) gene [18]. Normal ovarian function hinges on the presence of two copies of X-linked genes; the loss of one copy manifests as TS-related phenotypes. Recent research has additionally implicated telomere function, length, and epigenetic modifications in TS-related POI [19].

Trisomy X Syndrome (TXS), identified by a 47, XXX karyotype, is a sex chromosome aneuploidy disorder impacting approximately 1 in 1000 women, with a diagnostic rate of merely 10% [20]. Despite limited research, a 2020 study revealed diminished Anti-Mullerian Hormone (AMH) levels in TXS patients, suggesting an augmented risk of POI [21]. TXS individuals may also exhibit elevated FSH and Luteinizing Hormone (LH), correlating with menstrual cycle disorders and POI. Earlier investigations allude to a potential correlation between TXS and POI [22].

#### Structural chromosomal abnormality

In the realm of structural rearrangements involving the X chromosome, the isochromosome (46, Xi(X)(q10)) emerges as a prevalent occurrence, often associated with the TS phenotype, indistinguishable from 45, X patients. Deletions and translocations within the long arm of the X chromosome are also reported in cases of POI. Deletions typically exhibit breakpoints in the Xq24–Xq27 region, while translocation breakpoints predominantly occur from Xq13 to Xq21. These observations designate the Xq24–q27 and Xq13.1–q21.33 regions as POI critical regions 1 and 2, respectively [23].

In addition, the correlation between X-autosomal translocation and POI cannot be ignored. Various genes on the X chromosome have been implicated through X-autosomal translocations, encompassing Diaphanous Related Formin 2, Premature Ovarian Failure, 1B, Progesterone receptor membrane component 1, among others. Despite the rarity of such rearrangements (1:30,000), distinct patterns in the localization of X-chromosome breakpoints have been discerned – 80% of the breakpoints fall within the Xq21 cytoband of the POI2 region [24]. The correlation between X-autosomal translocation and POI remains inconclusive, with three prevailing hypotheses: Gene disruption, Meiosis error, and Position effect hypothesis [25].

While evidence substantiates the role of X chromosome abnormalities in POI, recent research in this domain has been limited. Analysis of data from four studies reveals a prevalence of X-chromosomal structural abnormalities and X-autosomal translocations in POI ranging from 4.2 to 12.0% [26]. This underscores the imperative for further exploration into the molecular and genetic mechanisms underpinning these abnormalities and their contribution to POI.

Apart from structural abnormalities in the X chromosome, Autosomal translocations, microdeletions, gene mutations, epistasis, and epigenetic changes linked to autosomal genes also constitute factors in POI. Previous studies have documented 28 cases of autosomal abnormalities associated with POI, encompassing 10 Robertsonian translocations, 10 reverse translocations, 5 chromosome inversions, and 3 autosomal chromosome microdeletions. These abnormalities manifest across diverse races, including Chinese, Thai, and American populations [27, 28].

### Gene mutation

#### Autoimmune disease-related POI

Autoimmune polyendocrine syndrome type 1 (APS-1) arises from mutations in the Autoimmune regulator (*AIRE*) gene, situated on 21q22.3 [29]. The *AIRE* gene, responsible for encoding a protein with two zinc fingers, functions as a transcription factor crucial for autoimmune tolerance. Its primary role lies in facilitating the expression of specific autoantigens in thymic stromal cells. Approximately 50 mutations in the *AIRE* gene have been identified, inherited through autosomal recessive transmission [30]. Notably, around 41% of APS-1 patients experience complications related to POI. The primary association between APS-1 and POI is autoimmune lymphocytic ovarian inflammation [31].

Ataxia-telangiectasia (AT) is a rare autosomal recessive genetic disorder that predominantly affects the nerves, blood vessels, skin, mononuclear macrophage system, and endocrine system, resulting in primary immunodeficiency [32]. The causative gene for AT is the Ataxia Telangiectasia Mutated (*ATM*) gene, which plays a crucial role in DNA damage repair, cell cycle regulation, and immune response, potentially influencing sexual maturity. The pathology of AT arises from the loss of protein expression due to *ATM* gene mutation. Clinical manifestations encompass cerebellar ataxia, ocular motility disorders, telangiectasia, immunodeficiency, tumor susceptibility, chromosome instability, and gonadal dysplasia. Female AT patients frequently present with ovarian hypoplasia and disorders in the development of primordial germ cells [33, 34].

#### Metabolic disease-related POI

Galactosemia is an autosomal recessive hereditary disorder arising from the deficiency of Galactose-1-Phosphate Uridylyltransferase (*GALT*) gene. This condition can result in severe complications in organs expressing high levels of GALT, including the liver, kidneys, ovaries, and heart [35]. POI manifests in 80-90% of female patients with homologous mutations in the *GALT* gene. *GALT* gene mutations can lead to the accumulation of galactose in the ovary, hindering its metabolism and inducing toxic effects, thereby fostering premature follicular atresia. The elevation of FSH in galactosemia patients initiates from birth to early adolescence, with varying onset times for ovarian function impairment [36]. However, primary amenorrhea predominates in most patients. Despite a minimal number of patients achieving natural conception, key biochemical indicators such as AMH, estradiol, and FSH consistently reflect ovarian failure [37].

Carbohydrate-Deficient Glycoprotein Syndrome is a congenital metabolic disorder stemming from impaired glycan binding to other complexes, such as proteins or lipids. The etiology of this syndrome is linked to a mutation in the Phosphomannomutase 2 gene. The Phosphomannomutase gene encodes a phosphomannose mutase responsible for catalyzing the conversion of mannose-6-phosphate to mannose-1-phosphate. A functional defect in this enzyme can disrupt ovarian glycoprotein glycosylation, potentially contributing to POI by affecting ovarian glucose metabolism [38].

#### Endocrine disease-related POI

Blepharophimosis-ptosis-epicanthus in-versus syndrome (BPES) is an autosomal dominant genetic disorder which manifests in two distinct types, with Type I commonly associated with POI, and its etiology correlated with mutations in the Forkhead transcription factor L2 (*FOXL2*) [39]. Over 260 *FOXL2* gene mutations have been found. These include changes in the polyalanine chain, leading to Type I or Type II BPES. *FOXL2* mutations can affect protein-protein or protein-DNA interactions [40]. However, some mutations don’t fully match the ovarian phenotype, especially missense mutations in the Forkhead domain [41]. Therefore, individuals with Type II BPES mutations may still need ovarian function monitoring.

Pseudohypoparathyroidism is a rare disorder, patients may encounter gonadal issues, such as delayed or impaired sexual development, absence of menstrual periods, irregular menstruation, or infertility. Approximately 70-80% of individuals with pseudohypoparathyroidism type 1a have a clearly identifiable genetic cause, predominantly attributed to GNAS Complex Locus **(***GNAS*) gene mutations or methylation changes [42]. The *GNAS* gene encodes the alpha subunit of the stimulatory heterotrimeric G protein, a critical component of the gonadotropin receptor signaling pathway. Individuals with gonadotropin resistance and POI may harbor mutant maternal *GNAS* alleles in the gonads, disrupting the gonadotropin receptor signaling pathway [43].

#### Nervous system disease-related POI

Ovarioleukodystrophy is a severe hereditary neurodegenerative disorder characterized by white matter loss and POI [44]. The condition is associated with mutations in the Eukaryotic initiation factor 2B (*EIF2B*) gene, a pivotal protein in the initiation of eukaryotic translation. EIF2B comprises five subunits: α, β, γ, δ, and ε. Mutations in any of these subunits can lead to the development of the disease. Individuals with mutations in the *EIF2B* gene often display progressive degenerative changes in the nervous system, and the severity of ovarian insufficiency is positively correlated with age [45]. However, in some cases, POI may manifest before the onset of neurological symptoms or during subclinical neurological lesions.

Progressive external ophthalmoplegia is characterized by a gradual onset of facial ptosis and restricted eye movement. Its pathogenesis is associated with mutations in the DNA Polymerase Gamma, Catalytic Subunit **(***POLG*) gene, which can manifest as either an autosomal dominant or recessive genetic disorder. This disorder falls within the category of mitochondrial diseases. Mutations in the *POLG* gene not only result in ocular symptoms but are also frequently accompanied by POI and Parkinson’s disease [46].

Perrault syndrome is a pleiotropic autosomal recessive disorder characterized by ovarian failure in females, sensorineural hearing loss in both genders, and neurological manifestations in select patients. In a European mixed-race family comprising two sisters, researchers detected compound heterozygous variants of HSD17B4 c.1704 A > G and c.17T > A [47]. Subsequent investigations revealed mutations in Histidyl-TRNA Synthetase 2, Mitochondrial **(***HARS2*), Leucyl-TRNA Synthetase 2, Mitochondrial **(***LARS2*), Caseinolytic Mitochondrial Matrix Peptidase Proteolytic Subunit **(***CLPP*), and Twinkle mtDNA Helicase **(***TWNK***)** within the Perrault syndrome framework, utilizing similar genomic methodologies such as linkage analysis or NGS, all crucial for maintaining normal mitochondrial function [48].

#### Other diseases-related POI

Premature aging syndromes, such as Bloom, Werner, and GAPO, are frequently associated with both female POI and male asthenospermia. These syndromes arise from specific gene mutations leading to autosomal recessive inheritance. Bloom syndrome, stemming from BLM RecQ Like Helicase gene mutations affecting DNA helicase, is characterized by short stature, a photosensitive rash, immunodeficiency, an elevated risk of tumors, and hypogonadism [49]; Werner syndrome, a typical human premature aging syndrome, results from WRN RecQ Like Helicase gene mutations. Symptoms encompass skin aging, scleroderma, cataracts, arteriosclerosis, a heightened risk of tumors, and gonadal dysplasia [50]; GAPO syndrome, caused by mutations in the ANTXR Cell Adhesion Molecule 1 gene involved in cell adhesion and migration, exhibits features such as growth retardation, alopecia, impacted teeth, optic nerve atrophy, and ovarian abnormalities in women [51]; Epidemiological research indicates a robust association between POI and premature aging syndromes. This suggests that POI can be considered a form of accelerated ovarian aging, even without obvious syndromic features.

Fragile X syndrome arises from a mutation in the Fragile X Messenger Ribonucleoprotein 1 (*FMR1*) gene on the X chromosome. The *FMR1* gene possesses a variable CGG repeat sequence, with a normal range of 6 to 44. When the repeats increase to 55 to 200, it is referred to as a premutation. Approximately 1 in 250 women carry the *FMR1* gene premutation, and 15–24% of them experience POI, known as Fragile X-related ovarian insufficiency (FXPOI). Studies indicate that about 11.5% of familial POI and 3.2% of sporadic POI patients harbor *FMR1* gene premutations. Therefore, *FMR1* gene premutations represent a primary genetic cause of POI [52]. The mechanism of FXPOI remains unclear, but it is suggested that an increased number of premutant CGG repeats hinder the normal binding of the *FMR1* gene product to the ribosome 40 S subunit. This affects the translation of the protein FMRP, leading to reduced FMRP expression and a subsequent rise in transcription factors, resulting in elevated *FMR1* gene mRNA levels [52]. FMRP protein is prominently expressed in germ cells and fetal ovaries, regulating the growth of oogonia and the initial ovarian size. Interestingly, FMRP expression is observed in granulosa cells (GCs) of mature follicles but not in primordial and primary follicles. This shift in expression highlights the role of FMRP in regulating follicular maturation. Abnormal FMRP expression directly impacts follicle and egg development. Moreover, the increase in CGG repeats can have a toxic effect on GCs, leading to increased follicular death [53].

## Genetic etiology of non-syndromic POI

**Fig. 2:**
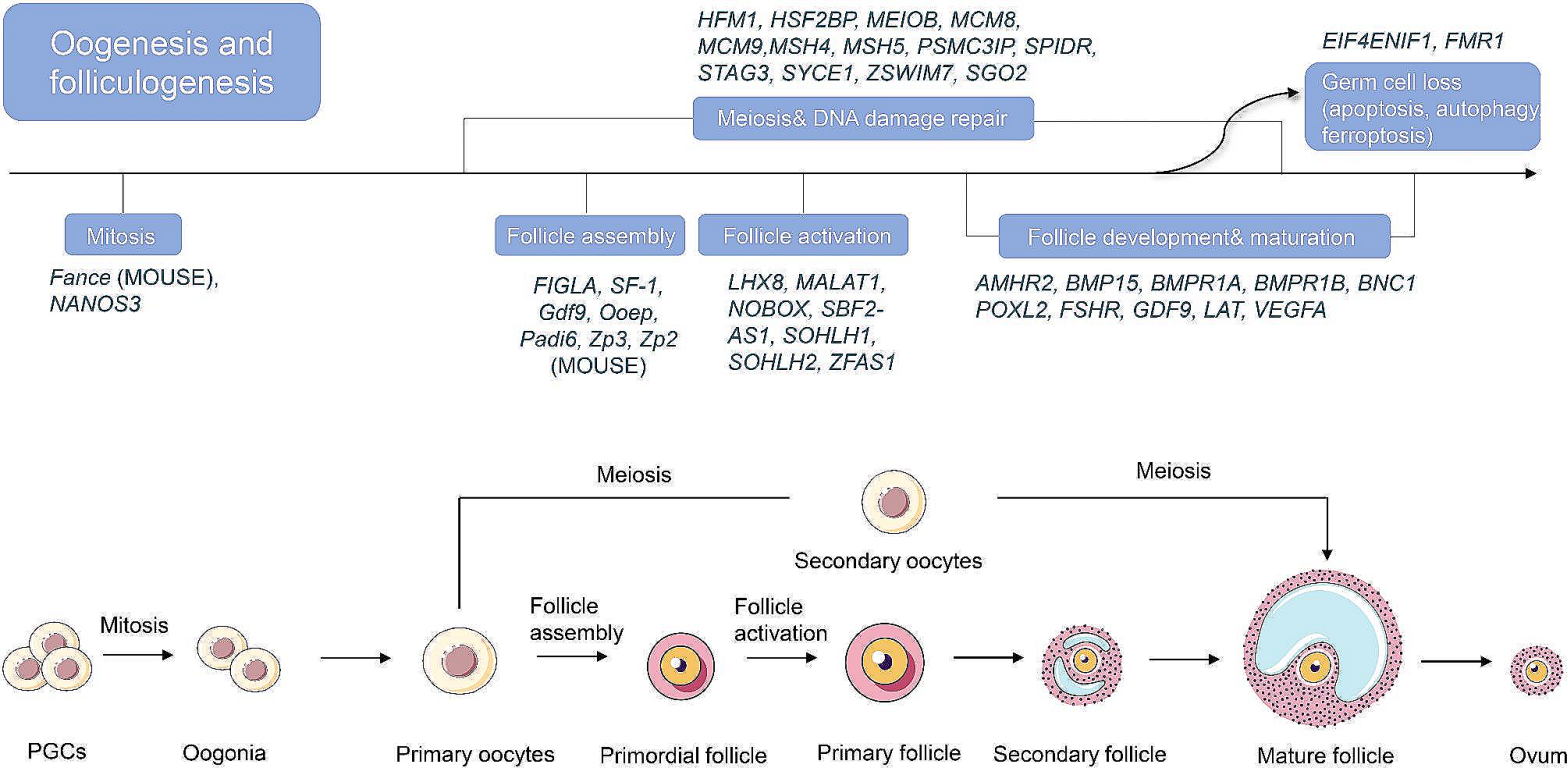
POI causative genes associated with oogenesis and folliculogenesis. The figure illustrates the correlation between pathogenic genes linked to POI and the developmental processes of oocytes and follicles. Initially, primordial germ cells undergo mitosis to differentiate into oogonia. These oogonia then enter meiosis, forming primary oocytes that pause at the diploid stage during early meiosis. Pregranulosa cells surround the oocytes during this phase, creating primordial follicles, which represent the dormant follicular reserve within the ovary. Oocytes lacking the encapsulation of primordial follicles may undergo apoptosis, resulting in their loss. The majority of primordial follicles remain quiescent, with only a minority undergoing activation. Once activated, these follicles undergo continuous growth, eventually either persisting in an atretic state or being released through ovulation in the later stages of follicular development. It is noteworthy that pathogenic genes associated with POI exert their influence across all stages of oocyte and follicular development, extending beyond the aforementioned developmental stages

Numerous genes are intimately associated with non-syndromic POI, implicating diverse biological processes. The involved processes and related genes are as followed. Homologous recombination and DNA damage repair: helicase for meiosis (*HFM1*), synaptonemal complex central element protein 1 (*SYCE1*), stromal antigen 3 (*STAG3*), mutS homolog 4 (*MSH4*), mutS homolog 5 (*MSH5*), etc. Follicle development and maturation: growth differentiation factor 9 (*GDF9*), *FOXL2*, *FSHR*, etc. Mitosis: nanos C2HC-type zinc finger 3 (*NANOS3*). Follicle assembly: folliculogenesis specific basic Helix-Loop-Helix gene A (*FIGLA*), SF-1. Follicle activation: LIM homeobox 8 (*LHX8*), neonatal ovarian homologous box (*NOBOX*), spermatogenesis and oogenesis specific basic Helix-Loop-Helix 1(*SOHLH1*), and oogenesis specific basic Helix-Loop-Helix 1 (*SOHLH2*). As well as cell death pathways: FMR1, and eukaryotic translation initiation factor 4E nuclear import factor 1 (*EIF4ENIF1*) (Table 1). These POI-associated genes exert their influence across all stages of oocyte and follicular development (Fig. 2).

**Table 1:** List of candidate genes implicated in non-syndromic POI

GENES	VARIANTS	ACMG pathogenic classification	Domain	Knockout mice phenotype	References
*HFM1*	c.3470G > A p. (Cys1157Tyr)	B	Nondomain	Deficient mice are sterile due to severe blockage of spermatogenesis and oogenesis.	[54, 144]
*STAG3*	c.3052delC p. (Arg1018AspfsTer14)	P	Nondomain	Deficient mice are sterile and their fetal oocytes are arrested at early prophase I leading to oocyte depletion at 1 week of age.	[61]
	c.659T > G p. (Leu220Arg)	LP	STAG domain		[61]
	c.3381_3384delAGAA p. (Glu1128MetfsTer43)	P	Nondomain		[70, 145]
*MCM8*	c.1561G > A p. (Asp521Asn)		MCM domain	Mice are viable but are sterile due to defects in double-strand break repair during gametogenesis. Ovaries are characterized by an early block of follicle development, and they later develop tumors.	[63, 146]
*MCM9*	c.911 A > G p. (Asn304Ser)	LB	MCM domain	Females are viable but are sterile due to defects in double-strand break repair during gametogenesis. Female ovaries are completely devoid of oocytes, and testes show a severe early proliferation defect of germ cells, causing a retarded development of only a fraction of seminiferous tubules that produce then apparently normal spermatozoa.	[63, 146, 147]
*MSH4*	c.2355 + 1G > A p. (Ile743_Lys785del)	P	ATPase domain of DNA mismatch repair MUTS family	NR	[72]
	c.2374 A > G p. (Thr792Ala)	LP	ATPase domain of DNA mismatch repair MUTS family		[73]
	c.2222_2225delAAGA p. (Lys741Argfs*2)	P	ATPase domain of DNA mismatch repair MUTS family		[73]
*MSH5*	c.1459G > T p. (Asp487Tyr)	VUS	DNA-binding domain of DNA mismatch repair MUTS family	increased circulating unsaturated transferrin level; abnormal lens morphology; increased leukocyte cell number; increased startle reflex	[74]
	c.1057 C > A p. (Leu353Met)	VUS	DNA-binding domain of DNA mismatch repair MUTS family		[74]
	c.2107 A > G, p. (Ile703Val)	VUS	ATPase domain of DNA mismatch repair MUTS family		[74]
*MEIOB*	c.1218G > A p. (Thr406=)	VUS	Nondomain	Mice develop and grow normally but show infertility in both sexes. Infertility is due to a meiotic arrest at a zygotene/pachytene-like stage. DNA double strand break repair and homologous chromosome synapsis are impaired in meiocytes.	[68]
	c.203del p. (Gly69AlafsTer19)	LP	Nondomain		[148]
	c.683-1G > A	LP	Nondomain		[148]
	c.258_259del p. (Val87AspfsTer3)?	LP	Nondomain		[69]
	c.1072_1073del p. (Met358ValfsTer12)	LP	Nondomain		[69]
	c.814 C > T p. (Arg272Ter)	P	Nondomain		[69, 149, 150]
*PSMC3IP*	c.206_208delAGA p. (Lys69del)	VUS	TBPIP/Hop2 winged helix domain	Infertility. Males exhibit testicular hypoplasia with lack of spermatozoa.	[151]
	c.189G > T p. (Lys63Asn)	VUS	TBPIP/Hop2 winged helix domain		[151, 152]
*HSF2BP*	c.500 C > Tp. (Ser167Leu)	VUS	Region 83–334 Interaction with BRCA2	Female mutants exhibit a fertility with 40% reduction in litter size compared to wild-type females.	[153]
	c.382T > C p. (Cys128Arg)	VUS	Region 83–334 Interaction with BRCA3		[153, 154]
*ZSWIM7*	c.173 C > G p. (Ser58Ter)	LP	Nondomain	NR	[82]
	c.231_232delAT p. (Cys78PhefsTer21)	P	Zinc finger		[155]
	c.38T > C p. (Leu13Pro)	VUS	Nondomain		[155]
*SPIDR*	c.839G > A p.(W280*)	P	Region 151-450Necessary for interaction with RAD51	female/male infertility; decreased bone mineral density; abnormal eye posterior chamber depth	[156]
*SYCE1*	c.613 C > T p. (Gln205Ter)	VUS	Coiled coil 52–290	female/male infertility; abnormal lens morphology; increased urine microalbumin level	[157]
*NOBOX*	c.1064G > A p. (Arg355His)	VUS	Homeobox domain	Male mice are fertile and show no obvious abnormality. Female mice lacking *Nobox* have normal gross anatomy and histology, but are infertile with atrophic ovaries that lack oocytes at 6 weeks of age.	[88]
	c.1079G > A p. (Arg360Gln)	B	Homeobox domain		[88, 157]
*FIGLA*	c.11 C > A p. (Ala4Glu)	B	Nondomain	Females display a defect in the formation of primordial follicles leading to infertility.	[92]
	c.625G > A p. (Val209Ile)	B	Nondomain		[92]
	c.84 C > A p. (Asp28Glu)	B	Nondomain		[92, 158]
*FSHR*	c.1268T > C p. (Ile423Thr)	VUS	G-protein coupled receptors family 1 profile	abnormal startle reflex; decreased bone mineral density; decreased lean body mass; increased fasting circulating glucose level;	[96]
*AMHR2*	c.626T > A p. (Ile209Asn)	LP	Domain 203–518 Protein kinase	NR	[159]
	c.1060 C > T p. (Leu354Phe)	VUS	Domain 203–518 Protein kinase		[159]
	c.50 C > A p. (Ala17Glu)	VUS	Nondomain		[159]
*BMP15*	c.791G > A p. (Arg264Gln)	VUS	Transforming growth factor-beta like domain	NR	[104]
	c.1076 C > T p. (Pro359Leu)	LP	Transforming growth factor-beta like domain		[104]
	c.309T > G p. (Asn103Lys)	LB	Nondomain		[106]
	c.551T > C p. (Met184Thr)	VUS	Nondomain		[106]
	c.406G > C p. (Val136Leu)	VUS	Nondomain		[105]
*LAT*	c.245 C > T p. (Pro82Leu)	LB	Nondomain	abnormal vitreous body morphology; increased circulating bilirubin level abnormal startle reflex	[107]
	c.181 C > G p. (Pro61Ala)	LB	Nondomain		[107]
*VEGFA*	c.1154G > A p. (Gly385Glu)	Uncertain Significance	Nondomain	NR	[108]
*EIF4ENIF1*	c.1286 C > A p. (Ser429Ter)	LP	Helicase C-terminal	small testis; edema; decreased circulating chloride level; abnormal urinary bladder morphology; abnormal embryo size	[111]
*BMPR1A*			Nondomain	NR	[95]
*BMPR1B*			Nondomain	small seminal vesicle; abnormal seminal vesicle morphology; female infertility small testis; abnormal testis morphology	[95]

Abbreviations: P, pathogenic; LP, likely pathogenic; VUS, variants of uncertain significance; LB: likely benign; B, benign; NR, no record. The lines pertaining to BMPRIA and BMPRIB denote investigations involving gene knockout mice, hence solely presenting the phenotypic profile of the knockout mice in this context

### Meiosis and DNA damage repair factors

An adequate oocyte reserve is imperative for women of reproductive age in ensuring the birth of healthy offspring. Nonetheless, gene mutations involved in meiosis, DNA replication, or DNA repair can result oogenesis abnormality and consequent infertility. Hence, a comprehensive investigation into the genes governing these key processes, is essential for an enhanced comprehension of POI.

#### Meiosis and DNA replication related-genes

*HFM1* plays a pivotal role in homologous chromosome crossover and synapsis completion during meiosis. A specific *HFM1* mutation (c.3470G > A) was identified in a Chinese family with POI, with bioinformatics tools suggesting its potential association with mRNA splicing [54]. *Hfm1* knockout mice exhibit decreased follicular reserve and fertility. Subsequent investigations suggest that HFM1 may contribute to the pathogenesis of POI by influencing spindle assembly and division [55].

Another key gene is *SYCE1* which encodes a constituent of the synaptonemal complex that mediates homologous chromosomes synapsis and promotes crossover formation [56]. In 2014, the first POI-associated *SYCE1* variant (c.613 C > T) was reported [57]. Mouse models carrying this variant exhibit infertility and significantly reduce *Syce1* translation levels [58]. In POI patients, *SYCE1* mutations have been revealed can disrupt the interfaces formed between SYCE1 and synaptonemal complex component six6 opposite strand transcript 1 [59].

*STAG3* as another member of the Synaptonemal Complex, facilitates the nuclear localization of meiotic recombination protein REC8 through direct interaction. Meiotic recombination protein REC8-STAG3-containing cohesin regulates chromosomal topology and maintains sister chromatid cohesion [60]. In 2019, whole-exome sequencing of a Caucasian family with POI identified a truncation mutation (c.3052delC) and a missense mutation (c.659T > G), both predicted to significantly affect domain structural integrity [61].

Co-expressed with *STAG3*, minichromosome maintenance 8 (*MCM8*) and minichromosome maintenance 9 (*MCM9*) likely share similar functions. MCM8/9 facilitates normal replication fork progression and protects stalled forks from excessive degradation [62]. In 2016 a patient with heterozygous mutations in both *MCM8* and *MCM9* was identified [63]. Knockout experiments in mice revealed *Mcm8* and *Mcm9* are crucial for gametogenesis and genomic stability [64]. Cells with homozygous MCM8 mutations exhibited significantly higher chromosomal breakage compared to controls when exposed to mitomycin C [65]. Recent research indicates that *MCM9* mutations reduce double-strand break (DSB) repair functionality [66].

Putative homologs of MCM8 found in other organisms include meiosis-specific with OB domain (MEIOB), a single-strand DNA-binding protein necessary for maintaining the appropriate number of RAD51 recombinase (RAD51) and DNA meiotic recombinase 1 (DMC1) foci after the zygotene stage. MEIOB forms a heterodimer with spermatogenesis associated 22 (SPATA22), interacting with the RPA heterotrimer, the primary complex in DNA metabolism [67]. In 2019, Caburet et al. reported the first POI-associated MEIOB mutation: c.1218G > A [68]. Recently, Wang et al. discovered two novel homozygous frameshift mutations (c.258_259del, c.1072_1073del) and a new homozygous nonsense mutation (c.814 C > T) in *MEIOB* [69]. These mutations disrupt theMEIOB-SPATA22 interaction [68, 69]. Moreover, *Meiob*-deficient mouse exhibit meiotic failure and infertility in both sexes [67].

Taken together, genes involved in meiosis and DNA replication related POI include *HFM1, SYCE1, STAG3, MCM8, MCM9, MEIOB*. *HFM1*mutations may cause POI by affecting spindle assembly and division [55]. As members of the synaptonemal complex, *SYCE1* and *STAG3* mutations might lead to POI [57, 61, 70]. Potential links between mutations in other complex components and POI require further exploration. MCM8 and MCM9, as members of the minichromosome maintenance family, regulate cell division, and mutations have been related with POI [63]. MEIOB forms a heterodimer with SPATA22 for proper homologous recombination, and experimental evidence demonstrates that POI-associated *MEIOB* mutations disrupt this interaction [68, 69]. Another gene such as PSMC3IP, its mutations have found in POI patients, but the pathogenic mechanisms remain unclear.

#### DNA repair related-genes

Mismatch repair systems, ubiquitous across organisms, are crucial for genomic stability. By rectifying base pair mismatches and small nucleotide indels during DNA replication and recombination, mismatch repair system ensures replication and recombination fidelity. *MSH4* and *MSH5*, as members of the DNA mismatch repair mutS family, encode meiosis-specific proteins. MSH4 and MSH5 heterodimers uniquely bind Holliday Junctions, stabilizing and preserving meiotic DSB repair intermediates [71]. Carlosama et al. identified a homozygous donor splicing site mutation (c.2355 + 1G > A) in *MSH4* in Colombian families with POI, predicted to inactivate the highly conserved Walker B motif in the ATP binding domain [72]. A recent study has suggested a correlation between *MSH4* variants and reduced oocyte quality by identifying biallelic *MSH4* variants in a diminished ovarian reserve patient [73]. For *MSH5*, Guo et al. reported a homozygous missense mutation (c.1459G > T) in Chinese sisters with POI, confirming its deleterious effects on DNA homologous recombination repair [74]. In 2020, Wang et al. proposed that through YB1, MSH5 and DNA damage repair transcriptionally regulated by the long non-coding RNA HCP5, implicating *MSH5* in the etiology of POI [75].

RAD51 and DMC1 play a pivotal role in DSB repair by facilitating the nucleoprotein filament to search for and invade its homologous chromosome [76]. Heat shock transcription factor 2 binding protein (HSF2BP) regulates RAD51/DMC1 localization at meiotic DSB sites through interaction with breast cancer type 2 susceptibility protein [77]. Felipe-Medina et al. reported a candidate missense variant of *HSF2BP* (c.500 C > T) in three families with POI. Further investigation revealed that 19 open reading frame 57 and MEIOB associated 1 (BRME1) serves as a potent interaction agent and stabilizer of HSF2BP. In meiotic cells carrying this mutation, staining of both HSF2BP and BRME1 reduced, along with fewer RAD51/DMC1 foci, suggesting a potential mechanism by which HSF2BP contributes to POI development [78].

During replication the Shu complex can regulate RAD51 recruitment to DNA repair foci. As a member of Shu complex, *SPIDR* plays a pivotal role in diverse processes, including homologous recombination regulation and RAD51 foci formation [79]. In 2017, a study reported a biallelic mutation in *SPIDR* associated with ovarian dysgenesis, altering SPIDR activity in homologous recombination [80]. Female mice lacking *Spidr* exhibit decreased fertility, while some *Spidr*^−/−^ oocytes still complete meiosis [81].

As mentioned above, *MSH4, MSH5, HSF2BP* and *SPIDR* are involved in DNA damage repair, participating in POI. *MSH4* and *MSH5* are key genes in DNA damage repair. In 2020, the pathogenic mechanism of POI-associated *MSH5* mutations was proposed [75], while the mechanism of POI-related MSH4 mutations requires further study. HSF2BP regulates the localization of RAD51/DMC1 [77], and recent studies suggest that its gene mutations, through interactor chromosome 19 open reading frame 57/BRME1, may lead to POI [78]. SPIDR and another member of Shu complex, zinc finger SWIM type containing 7, both of which have been found associated with POI [80, 82, 83]. Meiosis and DNA damage repair are critical processes during oogenesis, and genetic variations affecting these processes could lead to female reproductive abnormalities. In addition to the aforementioned genes, many other genes that potentially associated with POI, warrant further investigation.

### Transcription factor related-factors

Although transcription factors cannot directly participate in many important biological processes, they play a crucial role in regulating body activity by controlling various target genes. In female reproduction, they regulate the expression time, location, and level of reproductive genes to ensure the smooth progress of each reproductive process.

*SF-1* and Neonatal Ovarian Homologous Box (*NOBOX*) gene have been identified as the earliest transcription factor specifically involved in gonadotropin cells [84–86]. They play an important role in early ovarian development, including follicle assembly and growth maintaining the primordial follicle (PF) pool and oocyte-specific gene expression during early follicular development [84, 85]. In ovaries with *SF-1* deficiency, multiple key follicular related mechanisms are impaired. These damaged pathways lead to increased oocyte death and a significant decrease in ovarian reserve [84]. The absence of *SF-1* in the pituitary and GCs before and after ovulation indicates that this nuclear receptor plays an important role in the pituitary gonadal axis [85, 87].

In human gonads, *NOBOX* is preferentially expressed in primordial follicles and metaphase II oocytes [88]. A study of 96 white women with premature ovarian failure found two mutations c.1064G > A and c.1079G > A in the *NOBOX* gene that disrupted its DNA binding activity, leading to ovarian dysfunction [88]. Additionally, sumoylation has been shown to have an inhibitory effect on *NOBOX* transcriptional activation using Gdf9 as the promoter [89]. The function of Spermatogenesis and *SOHLH1* and *SOHLH2* is to coordinate oocyte differentiation without affecting meiosis and regulate the co expression of oocyte specific transcription regulatory factors. The function of *SOHLH1* and *SOHLH2 is* similar to *NOBOX* [90]. A study suggests that ubiquitination can directly regulate *SOHLH1* or serve as an indirect regulatory factor in *SOHLH2* localization [89]. A 2020 study explored *FIGLA*, *LHX8* and *SOHLH1*, showing that they are jointly involved in a multi-functional network, which plays an important role in regulating the maintenance and differentiation of germ cells during early mouse oogenesis [90].

*LHX8 is* expressed in germ cells and is crucial for early oogenesis. The global knockout and conditional knockout of *LHX8* leads to the primary follicle death and reduced reserve of secondary follicle pools; Another study found that *LHX8* ablation leads to DNA damage in oocytes, leading to a significant increase in autophagy and premature depletion of ovarian reserves. A study based on gene load testing identified LOF mutations in *LHX8*, characterized by delayed oocyte maturation, suggesting haploid dysfunction effects [91].

A study on Chinese patients with premature ovarian failure found three different *FIGLA* mutations c.11 C > A, c.625G > A and c.84 C > A in four patients. These mutations in FIGLA may affect the transcription of zona pellucida genes and cause ovarian dysfunction [92]. FIGLA is a key gene that influences the balance of other transcription factors. Without *FIGLA*, the expression of meiotic related genes (such as *SYCP3*, *RAD51*, *YBX2*) and oocyte growth/differentiation genes (like *NOBOX*, *LHX8*, *TAF4B*, *SOHLH1*, *SOHLH2*, *GDF9*) becomes imbalanced. The imbalance significantly affects meiosis, leading to DNA damage and oocyte apoptosis [90].

Transcription factors are pivotal in orchestrating various biological processes by controlling the expression of target genes. In female reproduction, they govern the timing, location, and levels of reproductive genes crucial for the smooth progression of reproductive processes. These findings of *SF-1*, *NOBOX*, *SOHLH1*, *SOHLH2* and *FIGLA* highlight the intricate regulatory network orchestrated by transcription factors in female reproductive health.

### Signal pathway

Signal molecules, such as hormones, have the ability to transmit information between cells and regulate cell activity by binding to receptors both inside and outside the cell. Multiple hormones and receptors are involved in many important processes related to female reproduction and fertility, and their expression levels in the human body can serve as indicators of female reproductive status.

*FSHR*, Anti-Müllerian Hormone Receptor Type II (*AMHR2*), Bone Morphogenetic Protein Receptor Type 1 A (*BMPR1A*) and Bone Morphogenetic Protein Receptor Type 1B (*BMPR1B*) are expressed in ovarian granulosa [93–95]. They play a crucial role in normal gonadal function, regulating follicle growth, estrogen production, and oocyte maturation. In patients with primary ovarian insufficiency and partial loss of FSHR function, folliculogenesis can proceed only up to the small antral stages. In addition, the complete inactivation of the FSHR in women may be caused by the absence of follicular recruitment during the reproductive life of these patients due to the inactive FSHR [94]. A 2020 study reported a novel missense mutation c.1268T > C in the *FSHR* gene in a female with normal puberty but primary amenorrhea, which severely affected cAMP/PKA signaling [96].

*AMHR2* is mainly expressed in GCs that grow follicles, and in the cumulus of large antral follicles. *AMHR2* mutations can trigger persistent Müllerian disorder syndrome in humans, characterized by the persistence of Müllerian tube derivatives and early failure of ovarian follicle banks. Other residues of *AMHR2* are also crucial for AMH signaling [97, 98]. Two new missense *AMHR2* variants (c.627T > A and c.1060 C > T) were found in a Chinese POI cohort. Mutations located in the intracellular domain, for instance c.627T > A, which is thought to disrupt the substrate-binding site of the kinase domain, migrate normally to the cell surface but are unable to transduce the AMH signal [99, 100].

BMPR1A or ALK3 is serine threonine kinase type I receptors expressed in many tissues. BMPR1B plays an important role in cumulus cell expansion, ovulation cycle, and skeletal system development. Ovarian specific Bmpr1a^−/−^ mice become infertile within 6 months after reproduction due to the cessation of follicular development. For Bmpr1b^−/−^ mice, the mutant exhibits cumulus expansion damage and endometrial development defects [95, 101].

*GDF9* and Bone Morphogenetic Protein 15 (*BMP15*) play a crucial role in follicle formation and development before the ovarian cycle. They form heterodimers or homodimers to activate primary follicles and participate in follicular development, ultimately mediating germ cell formation and ovulation processes [102, 103]. In 2014, high-resolution array comparative genomic hybridization (CGH) analysis revealed the first POI-related mutation that may affect the *GDF9* regulatory region [103]. A study found *BMP15* mutations c.791G > A and c.1076 C > T in two sisters with POI that affect the water molecules around the protein and the thermal stability [104]. Scientists also conducted in vitro cell line experiments and found that *BMP15* function was impaired. Two other studies also found harmful heterozygous *BMP15* variants c.309T > G, c.551T > C, and c.406G > C in POI patients [105, 106].

Linker for Activation of T Cells (*LAT*) and Vascular Endothelial Growth Factor A (*VEGFA*) gene mutation may be potential mechanisms for POI development. It is reported that a novel single compound heterozygous pathogenic variant of the *LAT* gene (c.245 C > T and c.181 C > G) has been discovered in a POI family, and for the first time, it has been reported that *LAT*, which was initially expressed in T cells, exists in ovarian GCs. These two variants reduce the expression of *LAT*, inhibit the proliferation of GCs, and promote GCs apoptosis, which may be related to the etiology of POI [107].

A study showed that *VEGFA* − 1154G > A and 936 C > T variants are linked to the susceptibility of Chinese Han women to primary ovarian insufficiency [108]. VEGFA essentially affects the ovarian folliculogenesis as well as the normal reproductive function. It has been found to lead to the extravasation of protein-rich fluid in ovarian follicles [109]. Hence, VEGFA genetic variants may be capable of modulating its expression and affecting POI.

Signal molecules, including hormones, orchestrate cell activity by binding to receptors, both inside and outside the cell. In female reproduction and fertility, numerous hormones and receptors are pivotal, serving as indicators of reproductive status. These findings of *FSHR*, *AMHR2*, *BMPR1A*, *BMPR1B* ,*GDF9*, *BMP15*, *LAT* and *VEGFA* underscore the intricate interplay between signaling molecules and receptors in regulating female reproductive processes and highlight their significance in POI.

### RNA metabolism and translation related-factors

RNA is closely related to DNA and proteins. Therefore, damaged RNA activity, including metabolism and translation, has adverse effects on proteins. These proteins may play important roles in various biological processes, such as follicle formation, steroid production, cell proliferation, and apoptosis.

In 2013, a study demonstrated that the c.457 C > T substitution decreases the stability of *NANOS3*, potentially resulting in a hypomorph. Furthermore, an investigation of the relationship between the number of primordial germ cells (PGCs) and the dosage of NANOS3 in mouse models showed that the population of PGCs is controlled by the level of NANOS3 protein [110]. There are three homologues: *NANOS1*, *NANOS2*, and *NANOS3*. Destroying *NANOS1* in mice does not affect germ cell development, but knocking out *NANOS2* or *NANOS3* can lead to infertility, resulting in a decrease in gonadal size due to the loss of PGCs [26].

In a large French Canadian family, 7 women with POI had heterozygous premature termination codons c.1286 C > A in *EIF4ENIF1*, which were not present in the unaffected members, indicating a dominant genetic pattern of POI leading to *EIF4ENIF1* mutations [111]. In addition, a study found a rare mutation in the *EIF4ENIF1* gene in a family with POI. This mutation may lead to mutations around the site α- Damage to the spiral structure or α- Reduction in spiral length. This mutation is the second new *EIF4ENIF1* mutation found in POI patients [112]. *EIF4ENIF1* is a factor that transports the translation initiation factor eIF4E. It competitively blocks the effective binding of eIF4E and eIF4G, thereby regulating ribosomal delay and reducing protein synthesis by interfering with their interaction [112].

RNA is intricately linked to DNA and proteins, and disruptions in RNA activity, including metabolism and translation, can have detrimental effects on protein function. These proteins are integral to various biological processes such as follicle formation, steroid production, cell proliferation, and apoptosis. These findings *NANOS3* and *EIF4ENIF1* highlight the critical role of RNA in regulating protein funtion and its implications for POI.

## Mitochondrial dysfunction-associated POI

**Fig. 3:**
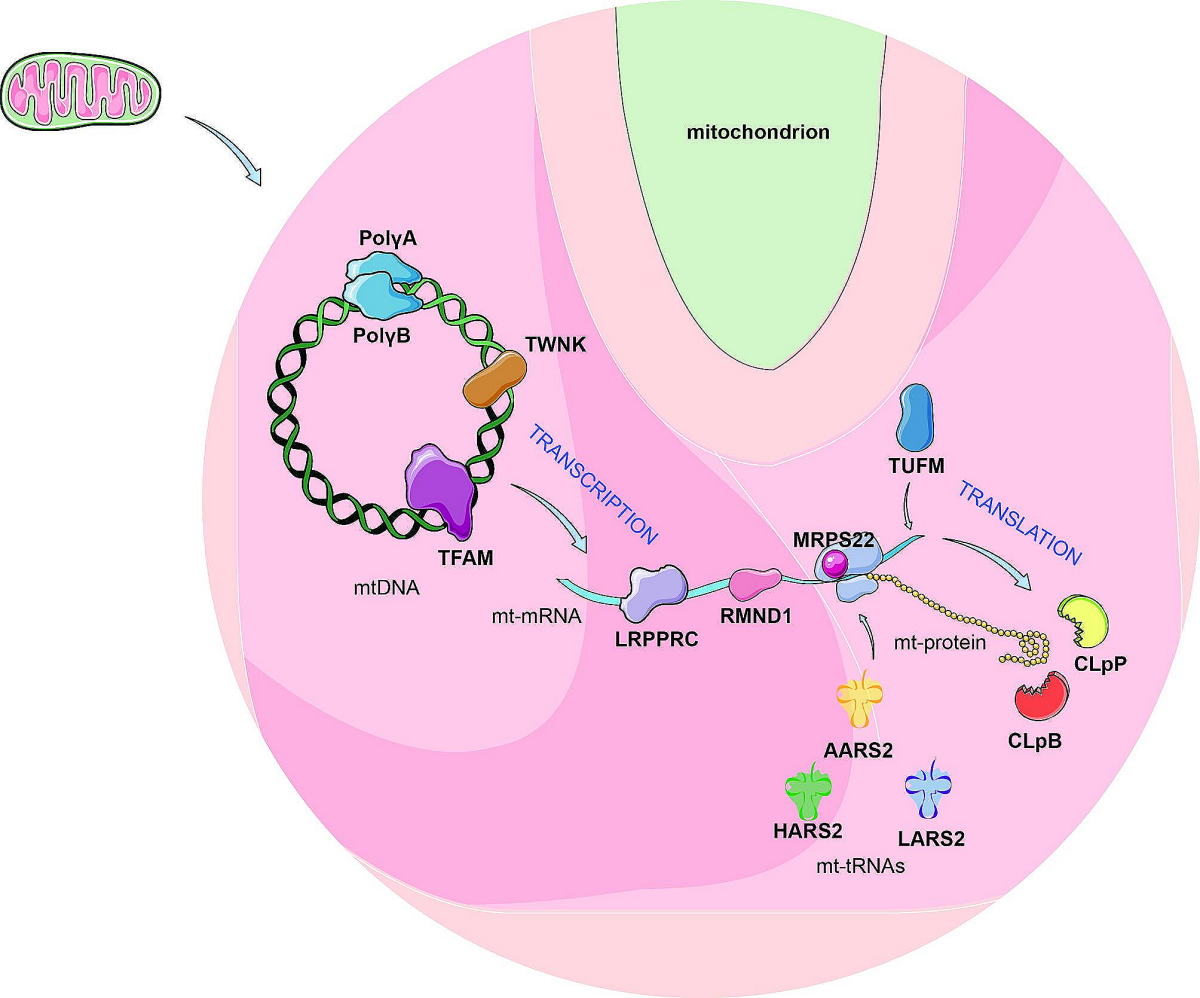
Schematic diagram of mitochondrial genes associated with POI. This figure illustrates the diverse molecular functions of proteins within mitochondria, which have been thoroughly characterized, establishing that mutations in these genes can lead to POI. The DNA polymerase γ (Polγ) consists of PolγA, encoded by the *POLG* gene, and PolγB, encoded by the *POLG2* gene. Additionally, TWNK encodes a mitochondrial helicase crucial for mtDNA replication processes. Together, they form the core machinery for mitochondrial DNA (mtDNA) replication. During transcription initiation, TFAM binds to mtDNA, activating transcription. MRPS22 is essential for the translation of mtDNA-encoded peptides and maintaining mitochondrial ribosomal complex stability. LRPPRC, an RNA-binding protein, enhances mRNA structure, translation accuracy, and stability, impacting the expression of both mitochondrial and nuclear genes. RMND1 contributes to mitochondrial translation, particularly in the synthesis of mtDNA-encoded peptides. The genes *HARS2*, *LARS2*, and *AARS2* encode aminoacyl-tRNA synthetases (aaRS), pivotal for translating mitochondrial-encoded genes. TUFM, a nuclear-encoded mitochondrial translation elongation factor, is crucial for mitochondrial protein synthesis. Moreover, CLpP and CLpB are involved in degrading damaged or misfolded mitochondrial proteins, maintaining mitochondrial health and function

Several genes associated with POI, whether in isolated instances or as part of syndromes, function within the mitochondria. These genes include Required for Meiotic Nuclear Division 1 homolog Gene (*RMND1*), Mitochondrial Ribosomal Protein S22 Gene (*MRPS22*), *POLG*, DNA Polymerase Gamma 2, Accessory Subunit (*POLG2*), Transcription Factor A, Mitochondrial (*TFAM*), Leucine-rich Pentapeptide Repeat Gene (*LRPPRC*), ClpB Family Mitochondrial Disaggregase (*CLPB*), *CLPP*, *TWNK*, Tu Translation Elongation Factor, Mitochondrial (*TUFM*), *LARS2*, Mitochondrial Alanyl-tRNA synthetase 2 (*AARS2*), and *HARS2* (Table 2). Broadly, their functions encompass mtDNA replication, transcription, translation as well as protein degradation (Fig. 3).

### mtDNA replication-related factors

The p140 catalytic subunit, PolγA (encoded by *POLG*), is a crucial component of DNA polymerase gamma (Polγ), responsible for replicating the mitochondrial genome. Mutations in POLG can lead to various mitochondrial diseases, including mitochondrial epilepsy, polyneuropathy, ataxia, and progressive external ophthalmoplegia [113]. Additionally, POI is an important clinical symptom resulting from *POLG* mutation. Previous studies typically describe POI caused by POLG mutations as part of a syndrome rather than isolated [114, 115]. However, in 2018, Chen et al. identified the first homozygous pathogenic *POLG* mutation, c.2890 C > T, causing non-syndromic POI through Whole exome sequencing (WES) [116]. Therefore, further studies are needed to establish a clear *POLG* genotype-phenotype correlation.

The genes encoding the four core components of mtDNA replicators are *POLG*, *POLG2*, *TWNK*, and *SSBP1*. Studies have confirmed that mutations in *POLG*, *POLG2*, and *TWNK* are associated with POI (Table 2). However, no mutations related to POI have been reported in the *SSBP1* gene, highlighting a need for further research in this area.

### mtDNA transcription-related factors

Mitochondrial RNA polymerase is primarily responsible for mtDNA transcription, requiring TFAM and mitochondrial transcription factor B2 (TFB2M) to initiate mtDNA transcription. TFAM plays a pivotal role in mtDNA transcription, maintenance, and replication. During transcription initiation, TFAM binds to mtDNA, bending it into a U-turn to activate transcription [117]. Mutations in *TFAM* are primarily associated with neurodegenerative diseases such as Alzheimer’s disease and Parkinson’s disease [118]. In 2020, Tucker et al. identified the most promising candidate mutation as a homozygous missense mutation, c.694 C > T, in *TFAM* while studying a patient with POI in Pakistan [119]. Subsequently, in 2021, Farid Ullah et al. confirmed the mutation’s pathogenicity through in vivo complementation studies in zebrafish [120].

Despite *TFAM* being identified as a strong candidate pathogenic gene for POI, its pathogenic mechanism remains unclear. Additionally, other genes involved in mtDNA transcription, particularly *TFB2M*, which shares functional similarities with *TFAM*, have not been reported to be associated with POI. Hence, further research in this area is warranted.

### Mitochondrial translation-related factors

Mammalian mitochondrial protein translation is a highly complex process divided into initiation, extension, termination, and circulation stages. It encompasses mitochondrial ribosome assembly, mRNA stability, and aminoacylation of amino acids with corresponding tRNA. Mutations in genes governing these processes impact translation, potentially causing various diseases [121]. Below, we discuss some gene mutations related to POI within this translation process.

The spectrum of diseases associated with *MRPS22* mutations ranges from isolated POI to severe mitochondrial disorders in infants [122]. MRPS22, a crucial component of the mitochondrial ribosomal 28 S small subunit, plays a key role in translating mtDNA-encoded peptides and maintaining mitochondrial ribosome complex stability [123]. A study by Chen et al. identified homozygous missense variants (c.404G > A and c.605G > A) in independent families and confirmed *MRPS22* mutations as an autosomal recessive cause of POI [124]. Isolated POI cases with *MRPS22* mutations do not typically exhibit other neurological or syndromic features, and the loss of *MRPS22* does not affect oxidative phosphorylation (OXPHOS), suggesting a non-energy-related mitochondrial function in POI [125].

LRPPRC, an RNA-binding protein, collaborating with SLIRP, it enhances mRNA structure, accuracy of translation, and stability, with distinct roles in mRNA polyadenylation and ribosomal guidance [126]. LRPPRC also interacts with eIF4e in the nucleus, impacting nuclear export and translation [127]. Mutations in *LRPPRC* lead to a rare disease known as Leigh syndrome, prevalent in the Saguenay-Lac-Saint-Jean region of Quebec and colloquially referred to as the French Canadian syndrome. Individuals with Leigh syndrome face a heightened risk of death due to neurological or acidosis crises triggered by factors such as infection, stress, diet, disease, or exercise. The average lifespan is less than 5 years, though some individuals may live longer. Female survivors may experience POI after puberty, characterized by elevated FSH levels, small ovaries with few follicles, and absence of menstrual periods [122]. Given *LRPPRC*’s impact on both mitochondrial and nuclear gene expression, further investigation is essential when considering its role in mitochondrial dysfunction-related POI.

In addition to *MRPS22* and *LRPPRC*, mutations in other genes that play a role in mitochondrial translation, including *RMND1*, *HARS2*, *LARS2*, *AARS2*, and *TUFM*, have also been shown to be associated with POI (Table 2)., emphasizing the importance of mitochondrial translation process for ovarian function. However, several unresolved issues persist: (i) the specific molecular mechanism underlying POI due to these gene mutations remains unclear; (ii) MRPS22 mutations have been reported in both isolated and symptomatic POI cases, establishing the genotype-phenotype relationship of MRPS22 is a key challenge; (iii) LRPPRC impacts both mitochondrial and nuclear gene expression. Whether its mutation causes POI by causing mitochondrial dysfunction remains to be confirmed.

### Protein degradation-related factors

Mammalian mitochondrial protein translation is a highly intricate process, categorized into four stages. One crucial protein is ClpB, also known as Suppressor of potassium transport defect 3 (SKD3), encoded by the *CLPB* gene and belonging to the AAA + family of ATPases. Located within mitochondria, ClpB plays a pivotal role in various activities, particularly in protein folding and the degradation of misfolded proteins. Despite its significance, its role in ovarian function remains unclear [128]. A study in 2022 revealed that female carriers of dominant ClpB pathogenic variants often experienced POI after puberty [129]. Another essential protein, ClpP, shares a similar function in degrading damaged or misfolded proteins. Encoded by the *CLPP* gene in humans, ClpP is a crucial component in forming endopeptidase Clp protein complexes [130]. In a study in 2013, recessive mutations of *CLPP* were identified in patients with Perrault syndrome, correlated with POI and sensorineural hearing loss, along with growth retardation. The disease’s pathological causes may involve insufficient clearance of mitochondrial components and inflammatory tissue damage [131]. Additionally, a novel variant of *CLPP* c.628G > A was discovered in a Chinese POI patient in 2023. Researchers also confirmed that CLPP deficiency impaired oxidative respiratory chain IV function by affecting COX5A, leading to reactive oxygen species (ROS) accumulation. This, in turn, is speculated to be the potential mechanism behind CLPP abnormalities accelerating ovarian follicular atresia, ultimately resulting in POI [132]. However, this hypothesis requires further investigation for validation.

In conclusion, the ClpB protein encoded by the CLPB gene and the ClpP protein encoded by the CLPP gene both contribute to protein degradation. Mutations in both the CLPB and CLPP genes have been linked to POI, although the exact pathogenic mechanism of CLPB mutations remains unclear. CLPP mutation may lead to POI by damaging the function of oxidative respiratory chain IV, leading to ROS accumulation, activating the intrinsic apoptotic pathway, and ultimately affecting the apoptosis of GCs. However, the hypothesis needs further verification.

## ncRNAs abnormalities in POI

Up to now, the pathogenic genes of POI have been extensively and deeply studied in the coding region. However, it is estimated that human protein coding sequences account for only 1.5% of the entire genome. ncRNA serves as an epigenetic marker regulating gene expression through interactions with DNA or RNA sequences and proteins. The primary categories of ncRNA include miRNAs and lncRNAs, both playing crucial roles in a diverse array of physiological and pathological processes. In recent years, more and more studies have shown that they are an important part of the genetic etiology of POI.

### Role of miRNAs in POI

MicroRNAs (miRNAs) have been implicated in an array of reproductive disorders, including POI, through the regulation of fertility-related genes. Despite this, the exact pathogenic mechanisms underlying their involvement remain poorly understood. Recent studies have shed light on the role of aberrantly expressed miRNAs in influencing GCs by modulating apoptosis and proliferation via specific signaling pathways.

Zhang and colleagues performed a comparative miRNA profiling analysis across multiple mammalian species, which highlighted the critical roles played by certain miRNA families and clusters in GCs apoptosis and follicular atresia. These include the let-7 family, miR-23-27-24 cluster, miR-183-96-182 cluster, and miR-17-92 cluster along with their corresponding pathways [133]. In findings from both Zhang et al. and Dang et al., it was reported that miR-127-5p and miR-379-5p exhibit significantly elevated expression levels in women with biochemically diagnosed POI. Overexpression of these miRNAs is correlated with suppressed proliferation of mouse GCs and a decline in DNA repair mechanisms, achieved by targeting key genes such as high mobility group box 2 (Hmgb2), poly ADP-ribose polymerase 1 (Parp1), and X-ray repair cross-complementing 6 (Xrcc6) [134, 135]. Separately, Chen et al. conducted a study where they identified a set of differentially expressed miRNAs in the plasma of POI patients. This set comprised ten upregulated miRNAs (miR-202, miR-146a, miR-125b-2*, miR-139-3p, miR-654-5p, miR-27a, miR-765, miR-23a, miR-342-3p, and miR-126) and two downregulated miRNAs (let-7c and miR-144). Notably, miR-146a was found to facilitate GCs apoptosis by directly targeting interleukin-1 receptor-associated kinase 1 (IRAK1) and tumor necrosis factor receptor-associated factor 6 (TRAF6), thus activating the caspase cascade pathway [136]. In another investigation by Yang et al., miR-23a was shown to induce apoptosis in human GCs by suppressing the expression of the anti-apoptotic protein XIAP and upregulating the cleaved, pro-apoptotic form of caspase-3 [137]. Furthermore, miR-23a also reduced the expression of SIRT1 and augmented apoptosis through the extracellular signal-regulated kinase 1/2 (ERK1/2) pathway [138].

These findings collectively suggest that miRNAs may serve as potential biomarkers and therapeutic targets for understanding and treating reproductive disorders like POI, given their regulatory influence on GCs function via modulation of apoptosis and proliferation pathways.

### Role of lncRNAs in POI

Two distinct mechanisms have been proposed to elucidate the role of long non-coding RNAs (lncRNAs) in POI. Firstly, the interaction between lncRNAs and miRNAs may contribute to the induction of apoptosis in ovarian GCs. For instance, Duan et al.‘s study revealed that the lncRNA DDGC stabilizes RAD51 mRNA through competitive binding to miR-589-5p, thus preventing ubiquitin-mediated degradation of the WT1 protein via interaction with heat shock protein 90 (HSP90),this mechanism impacts the DNA damage repair capability and differentiation of GCs [139]. Luo et al.‘s study demonstrated that the lncRNA HOTAIR acts as a molecular sponge for miR-148b-3p, relieving the inhibition of ATG14 and promoting autophagy activity and cell proliferation in GCs. Under normal physiological conditions, HOTAIR maintains appropriate levels of autophagy and GCs proliferation by regulating the miR-148b-3p/ATG14 axis. However, in the case of POI, down-regulation of HOTAIR may lead to autophagy disorders and impaired GCs function [140]. Another study by Zhang et al. observed a decreased expression of translation regulatory long non-coding RNA 1 (TRERNA1) in POI patients, suggesting its potential to sponge miR-23a and suppress GCs apoptosis [141].

Secondly, certain lncRNAs are implicated in regulating critical proteins and signaling pathways. For example, Wang et al.‘s study revealed that the lncRNA HCP5 plays a crucial role in stabilizing the binding of YB1 to the promoter region of the MSH5 gene, consequently enhancing the transcription and expression of MSH5. Additionally, HCP5 acts as an RNA scaffold, facilitating the interaction between YB1 and ILF2, which promotes the transport of YB1 into the nucleus. This timely regulation effectively modulates the function of MSH5 at DNA damage sites. Notably, the down-regulation of HCP5 expression in follicular GCs of POI patients impairs DNA damage repair mechanisms and increases GCs apoptosis [75]. Duan et al.‘s study found that lncRNA ZNF674-AS1 was down-regulated in follicular GCs of patients with biochemical ovarian insufficiency and was associated with serum levels of clinical ovarian reserve indicators. Further studies revealed that decreased expression of ZNF674-AS1 reduces aldolase A (ALDOA) enzyme activity and promotes the interaction between ALDOA and the proton pump (v-ATPase), thereby activating lysosomal-located adenylate-activated protein kinase (AMPK) and regulating glycolysis and GCs proliferation [142]. Another study by Duan et al. showed that lncRNA GCAT1 regulates CDKN1B (p27) translation by competitively binding to polypyrimidine tract-binding protein 1 (PTBP1) in GCs. In POI patients, down-regulation of GCAT1 inhibits G1/S cell cycle progression in GCs, thereby inhibiting GCs proliferation [143].

In conclusion, the above studies highlight the intricate roles of various lncRNAs in regulating key processes such as apoptosis, autophagy, DNA damage repair, and cell cycle progression in GCs, shedding light on underlying molecular mechanisms of POI.

## Conclusions, discussions, and perspectives

Currently, research on POI has identified numerous relevant genetic factors. This literature review synthesizes genetic factors and potential mechanisms related to POI across four dimensions: syndrome-associated POI, non-syndrome-associated POI (Table 1), mitochondrial dysfunction-associated POI (Table 2), and non-coding RNA-associated POI. The discussion delves into the intricate relationship between these genes and ovarian development, elucidating the functional consequences of diverse mutations to underscore the fundamental impact of genetic effects on POI. These insights serve to consolidate and enhance our comprehension of POI’s etiology, providing a theoretical basis for the diagnosis and treatment of POI patients and exploration of the disease mechanism.

**Table 2:** List of candidate genes implicated in mitochondrial dysfunction-associated POI

GENES	VARIANTS	ACMG pathogenic classification	Domain	Knockout mice phenotype	References
*RMND1*	c.713 A > G p. (Asn238Ser)	P	Domain of unknown function DUF155 226–403	NR	[160]
	c.583G > A p. (Gly195Arg)	VUS	Nondomain		[161]
	c.818 A > C p. (Tyr273Ser)	LP	Domain of unknown function DUF155 226–403		[161]
*MRPS22*	c.605G > A p. (Arg202His)	LP	Small ribosomal subunit protein mS22 21–359	Heterozygous MRPS22 knockout mice are fertile and show no overt abnormalities. Homozygous MRPS22 knockout results in embryonic lethality.	[124]
	c.404G > A p. (Arg135Gln)	VUS	Small ribosomal subunit protein mS22 21–359		[124]
*POLG*	c.2890 C > T p. (Arg964Cys)	VUS	Nondomain	premature aging and reduced lifespan.	[116, 162]
*POLG2*	c.1297G > T p. (Asp433Tyr)	LP	Picornavirus capsid 359–522	Heterozygous knockout mice are haplosufficient and develop normally; homozygous knockout mouse is embryonic lethal at day 8.0–8.5 p.c. with concomitant loss of mtDNA and mtDNA gene products.	[163, 164]
*TFAM*	c.694 C > T p. (Arg232Cys)	VUS	HMG-box_TFAM_rpt2 135–234	Embryonic lethal, due to absence of mitochondrial DNA. Mutant embryos die before 10.5 dpc.	[119, 165]
*LRPPRC*	c.1061 C > T p. (Ala354Val)	P	Nondomain	Loss of hepatic LRPPRC results in growth delay, and pronounced liver histopathological abnormalities; Loss of LRPPRC induces defective respiration under phosphorylating conditions.	[166–168]
*CLPB*	c.1159 C > T p. (Arg387Ter)	P	AAA + ATPase domain 373–527	NR	[129]
	c.1257 + 5G > A p.?	LP	AAA + ATPase domain 373–527		[129]
*CLPP*	c.628G > A p. (Ala210Thr)	VUS	CLP-protease 68–246	Homozygous pups are born at about 60% of the expected Mendelian rate, indicating decreased intrauterine survival. Mutant mice are smaller in size than wild-type littermates, show decreased motor activity, are completely deaf after 12 months and their lifespan is decreased relative to that of wild-type littermates. Both female and male mutant mice are completely infertile due to defects in germ cell development.	[132, 169]
*TWNK*	c.1172G > A p. (Arg391His)	VUS	SF4 helicase domain 384–635	NR	[47]
	c.1754 A > G p. (Asn585Ser)	LP	SF4 helicase domain 384–635		[47]
	c.1519G > A p. (Val507Ile)	VUS	SF4 helicase domain 384–635		[47]
	c.1321T > G p. (Trp441Gly)	LP	SF4 helicase domain 384–635		[47]
*HARS2*	c.1010 A > G p. (Tyr337Cys)	VUS	Aminoacyl-tRNA synthetase, class II 62–417	Disruption of Hars2in Cochlear Hair Cells Causes Progressive Mitochondrial Dysfunction and Hearing Loss in Mice.	[48, 170]
*LARS2*	c.1565 C > A p. (Thr522Asn)	LP	Aminoacyl-tRNA synthetase, class Ia 444–600	NR	[48]
	c.1077delT p.?	P	Nondomain		[48]
	c.1886 C > T p. (Thr629Met)	VUS	Nondomain		[48]
*TUFM*	c.524G > C p. (Gly175Ala)	VUS	Translational (tr)-type GTP-binding domain 58–254	NR	[171]
*AARS2*	c.149T > G p. (Phe50Cys)	LP	Alanyl-tRNA synthetase, class IIc, core domain 37–792	NR	[172]
	c.1561 C > T p. (Arg521Ter)	P	Alanyl-tRNA synthetase, class IIc, core domain 37–792		[172]

Abbreviations: P, pathogenic; LP, likely pathogenic; VUS, variants of uncertain significance; LB: likely benign; B, benign; NR, no record

However, several challenges persist in genetic research on POI patients. Firstly, the detailed mechanisms of action of many pathogenic genes and the specific interactions between different genes in the context of POI are not yet fully elucidated. Moreover, the specific mechanisms of pathways related to meiosis, such as DNA damage repair pathways, and their role in the development of POI remain unclear. It is also uncertain whether other genes involved in meiotic pathways can contribute to the development of POI. Transcription factor genes are known to play critical roles in early ovarian development, with their balance regulated by *FIGLA*. However, the precise interactions among these genes are not completely understood. The binding of signaling molecules to receptors is crucial for guiding follicle development. For instance, genes like *BMP15* and *GDF9* exhibit moderate expression levels in primary and early follicular populations, but their expression dramatically increases in mid-follicles. Nevertheless, the exact mechanism of interaction between *BMP15* and *GDF9* remains elusive [102].

Secondly, findings from animal experiments may not directly translate to humans. The differences in the gonadotropin-dependent stage between humans and rodents suggest that results obtained from animal models may not be directly applicable to humans due to genetic and physiological differences [94]. Furthermore, mouse models may not fully replicate the intricate interactions between molecules, cells, organs, organisms, and the environment.

There are several unresolved issues in current research on mitochondria and POI. For example, while certain gene mutations can lead to POI as mentioned in the text, mutations in other mitochondria genes with similar functions do not seem to have the same effect. The underlying reasons for this discrepancy remain unclear. Furthermore, the mechanism by which mutations in mitochondria genes can lead to a spectrum of diseases, ranging from isolated POI to multi-organ syndrome diseases, is not well understood. Additionally, there is a question of whether we can predict genotype-phenotype correlations in these cases.

Lastly, due to the heterogeneity of POI and the various modes of mutation transmission, the prevalence and types of genetic mutations vary significantly across different regions and races. Therefore, future genetic research should consider population stratification to accurately analyze genetic changes. It is crucial to involve different ethnic groups and larger sample sizes in future studies. Clinically, caution should be exercised when applying data from different racial groups in consultations.

In conclusion, genetic factors primarily affect ovarian development and follicle formation in POI, but the specific mechanisms of many gene variations and their effects are still unclear. Future research could focus on investigating ovarian development, follicle formation processes, and the functions of various genes at different stages of follicle development. Additionally, the association between POI and mitochondria, as well as non-coding RNAs, is a promising research area that will require further attention. These efforts aim to enhance clinical understanding and diagnostic approaches for POI.

## Data Availability

No datasets were generated or analysed during the current study.

## Declarations

### Author information

Linhang Nie, Xiaojie Wang, and Songyuan Wang contributed equally to this work.

## Abbreviations

POI: Premature Ovarian Insufficiency
FSH: Follicle-stimulating hormone
GCs: Germ cells
PGCs: Primordial germ cells
ncRNAs: Non-coding RNAs
HFM1: Helicase for meiosis
SYCE1: Synaptonemal complex central element protein 1
STAG3: Stromal antigen3
MCM8: Minichromosome maintenance 8
MCM9: Minichromosome maintenance 9
DSB: Double-strand break
MEIOB: Meiosis-specific with OB domain
RAD51: RAD51 recombinase
DMC1: DNA meiotic recombinase 1
SPATA22: Spermatogenesis associated 22
PSMC3IP: PSMC3 interaction protein
MSH4: MutS homolog 4
MSH5: MutS homolog 5
HSF2BP: Heat shock transcription factor 2 binding protein
BRME1: MEIOB associated 1
ZSWIM7: Zinc finger SWIM-type containing 7
SPIDR: Scaffold protein involved in DNA repair
NOBOX: Neonatal Ovarian Homologous Box
SOHLH1: Spermatogenesis and Oogenesis Specific Basic Helix-Loop-Helix 1
SOHLH2: Spermatogenesis and Oogenesis Specific Basic Helix-Loop-Helix 2
FIGLA: Folliculogenesis Specific Basic Helix-Loop-Helix Gene A
LHX8: LIM homeobox 8
AMHR2: Anti-Müllerian Hormone Receptor Type II
BMPR1A: Bone Morphogenetic Protein Receptor Type 1 A
BMPR1B: Bone Morphogenetic Protein Receptor Type 1B
GDF9: Growth Differentiation Factor 9
BMP15: Bone Morphogenetic Protein 15
LAT: Linker for Activation of T Cells
VEGFA: Vascular Endothelial Growth Factor A
TCR: T cell antigen receptor
NANOS3: Nanos C2HC-type zinc finger 3
EIF4ENIF1: Eukaryotic translation initiation factor 4E nuclear import factor 1
TS: Turner Syndrome
TXS: Trisomy X Syndrome
SHOX: Short stature homeobox
AMH: Anti-Mullerian Hormone
LH: Luteinizing Hormone
APS-1: Autoimmune polyendocrine syndrome type 1
AIRE: Autoimmune regulator
AT: Ataxia-telangiectasia
ATM: Ataxia Telangiectasia Mutated
GALT: Galactose-1-Phosphate Uridylyltransferase
BPES: Blepharophimosis-ptosis-epicanthus in-versus syndrome
FOXL2: Forkhead transcription factor L2
SF-1: Steroidogenic factor 1
GNAS: GNAS Complex Locus
EIF2B: Eukaryotic initiation factor 2B
POLG: DNA Polymerase Gamma, Catalytic Subunit
POLG2: DNA Polymerase Gamma 2, Accessory Subunit
HARS2: Histidyl-TRNA Synthetase 2, Mitochondrial
LARS2: Leucyl-TRNA Synthetase 2, Mitochondrial
CLPP: Caseinolytic Mitochondrial Matrix Peptidase Proteolytic Subunit
TWNK: Twinkle mtDNA Helicase
FMR1: Fragile X Messenger Ribonucleoprotein 1
FXPOI: Fragile X-related ovarian insufficiency
RMND1: Required for Meiotic Nuclear Division 1 homolog
MRPS22: Mitochondrial Ribosomal Protein S22
TFAM: Transcription Factor A, Mitochondrial
CLPB: ClpB Family Mitochondrial Disaggregase
TUFM: Tu Translation Elongation Factor, Mitochondrial
AARS2: Mitochondrial Alanyl-tRNA synthetase 2
WES: Whole exome sequencing
aaRSs: Aminoacyl-tRNA synthetases
